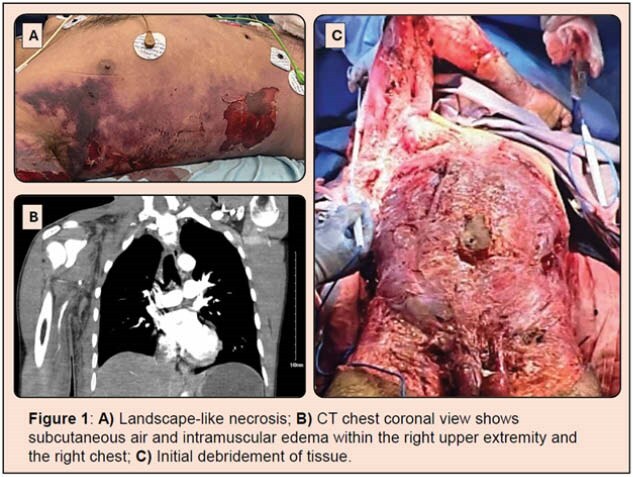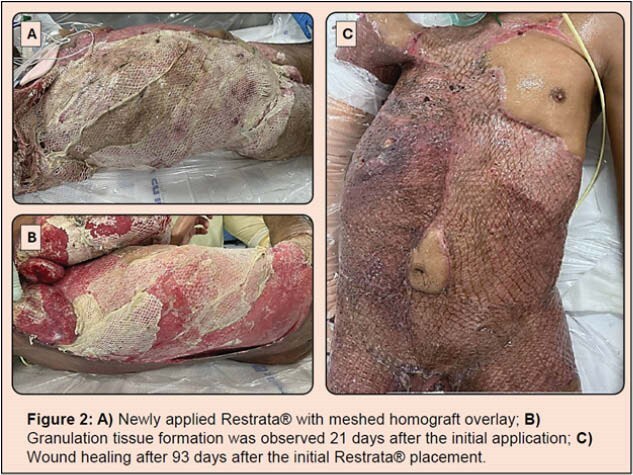# 843 Synthetic Electrospun Fiber Matrix Utilized in Conjunction with Homograft Wound Dressings in the Management of Post Excisional Massive Necrotizing Fasciitis: A Case Report

**DOI:** 10.1093/jbcr/iraf019.374

**Published:** 2025-04-01

**Authors:** Cherry Song, Michael Marano, Robin Lee, Christina Lee, Mostafa Elbahrawy, Kathryn Bregna, Dylan Brion

**Affiliations:** Cooperman Barnabas Medical Center; Cooperman Barnabas Medical Center; Cooperman Barnabas Medical Center; Cooperman Barnabas Medical Center; Community Medical Center; Rutgers New Jersey Medical School; Cooperman Barnabas Medical Center

## Abstract

**Introduction:**

Necrotizing fasciitis (NF) is an aggressive bacterial infection of the skin and subcutaneous tissues [1]. Urgent surgical intervention is necessary to debride necrotic and infected tissues, leaving large, open wounds. In this case, a patient presenting with NF underwent soft tissue debridement. A resorbable, fully synthetic electrospun fiber matrix (SEFM) was utilized to stimulate granulation tissue in a critically ill patient [2].

**Methods:**

A 39-year-old male presented with aggressive NF. Culture demonstrated Group A Streptococcus. Infected and necrotic tissues were surgically debrided across the full torso, right axilla, right upper arm and forearm, bilateral flanks, bilateral groins, bilateral anterior thighs, scrotum, and base of the penis, resulting in an excised area of 4,832 cm² with exposed structures. Total body surface area (TBSA) was 32%. The patient was transferred to a verified Burn Center for rapid initiation of wound care. SEFM treatment was initiated with standard wound care to stimulate granulation tissue formation in preparation for split-thickness skin grafting (STSG).

**Results:**

Meshed SEFM sheets were applied to the wound area and dressed with abdominal pads during the initial operation. To maintain contact between the SEFM and wound bed and to control for fluid loss, reapplication of SEFM with homograft overlay was performed in the operating room (OR) in sections over 35 days. The patient remained intubated in the supine position and underwent regular wound cleaning with chlorhexidine, sodium hypochlorite solution, and baby shampoo while the SEFM resorbed.

Granulation tissue was observed starting day 21 after initial application. On day 35, granulation tissue covered the left torso and bilateral thighs. The patient returned to the OR for the first of many STSG procedures. Following debridement with a saline jet system, a STSG was harvested from the patient’s left thigh. The 4:1 meshed STSG was applied to the left torso in with meshed homograft overlay. This process was repeated on the back, right torso, right upper thigh, right arm, right axilla, and genitalia. On day 63, the patient was alert and walking with assistance. A 95% graft take was observed in grafted regions, with minor graft loss due to location and body position. As of day 123, only minor openings remain.

**Conclusions:**

SEFM was utilized to stimulate granulation tissue formation for subsequent skin grafting over a massive area of tissue loss. Homograft overlay was utilized to limit fluid loss and maintain SEFM contact with the wound. As a synthetic, SEFM resisted enzymatic degradation within the wound despite frequent cleanings. SEFM may provide a novel solution in managing NF debridement wounds.

**Applicability of Research to Practice:**

The novel approach presented here shows promise in managing massive areas of soft tissue loss.

**Funding for the Study:**

No external funding was received.