# Correction to: Synthetic Electrospun Fiber Matrix Utilized in Conjunction with Homograft Wound Dressings in the Management of Post Excisional Massive Necrotizing Fasciitis: A Case Report

**DOI:** 10.1093/jbcr/iraf055

**Published:** 2025-05-16

This is a correction to: Cherry Song, Michael Marano, Robin Lee, Christina Lee, Mostafa Elbahrawy, Kathryn Bregna, Dylan Brion, 843 Synthetic Electrospun Fiber Matrix Utilized in Conjunction with Homograft Wound Dressings in the Management of Post Excisional Massive Necrotizing Fasciitis: A Case Report, *Journal of Burn Care & Research*, Volume 46, Issue Supplement_1, March/April 2025, Page S286, https://doi.org/10.1093/jbcr/iraf019.374

In the originally published online version of the manuscript the title had been changed to read: “Synthetic Electrospun Fiber Matrix Utilized in Conjunction with Homograft Wound Dressings in the Management of Post Excisional Massive Necrotizing Fasciitis: A Case Report” instead of: “Management of Massive Necrotizing Fasciitis Excision Utilizing a Synthetic Electrospun Fiber Matrix and Homograft Overlay”. The PDF version of the article is now similarly emended.

